# A Wearable Biofeedback Device to Increase Gait Swing Time Could Have Positive Effects on Gait among Older Adults

**DOI:** 10.3390/s22010102

**Published:** 2021-12-24

**Authors:** Alexandra Giraldo-Pedroza, Winson Chiu-Chun Lee, Wing-Kai Lam, Robyn Coman, Gursel Alici

**Affiliations:** 1 School of Mechanical, Materials, Mechatronic and Biomedical Engineering, Faculty of Engineering and Information Sciences, University of Wollongong, Wollongong, NSW 2522, Australia; jagp638@uowmail.edu.au (A.G.-P.); gursel@uow.edu.au (G.A.); 2 Applied Mechatronics and Biomedical Engineering Research (AMBER) Group, University of Wollongong, Wollongong, NSW 2522, Australia; 3 Li Ning Sports Science Research Center, Beijing 101111, China; 4 Department of Kinesiology, Shenyang Sport University, Shenyang 110102, China; 5 School of Health and Society, Faculty of Arts, Social Sciences & Humanities, University of Wollongong, Wollongong, NSW 2522, Australia; rcoman@uow.edu.au; 6 ARC Centre of Excellence for Electromaterials Science, University of Wollongong Innovation Campus, North Wollongong, NSW 2500, Australia

**Keywords:** wearable device, biofeedback, gait, elderly, biomechanics, walking ability, gait retraining

## Abstract

Older adults walk with a shorter stride length, reduced hip range of motion (ROM) and higher cadence. These are signs of reductions in walking ability. This study investigated whether using a wireless smart insole system that monitored and provided biofeedback to encourage an extension of swing time could increase stride length and hip flexion, while reducing the cadence. Seven older adults were tested in this study, with and without the biofeedback device, in an outdoor environment. Gait analysis was performed by using GaitRite system and Xsens MVN. Repeated measures analysis demonstrated that with biofeedback, the swing time increased by 6.45%, stride length by 4.52% and hip flexion by 14.73%, with statistical significance. It also decreased the cadence significantly by 5.5%. This study has demonstrated that this smart insole system modified positively the studied gait parameters in older adults and has the potential to improve their walking ability.

## 1. Introduction

Walking is a daily activity that has been found to reduce the risk of geriatric mortality [1]. However, aging induces gait modifications and one noticeable change is related to shorter stride length [2]. Reduction in stride length has been associated with deterioration in the general health of older adults [2,3,4,5,6,7,8,9]. In addition, older adults with significantly shorter stride length are less willing to walk [10] and have more walking instability [11,12]. Evidence has suggested that reduced hip range of motion reduces step length [13,14,15]. In particular, reductions in hip flexion during the late swing can reduce the length that each step can travel [16]. To compensate for the shorter stride length, researchers have found that older adults would increase their cadence to match younger adults’ velocity [17]. Nevertheless, increased cadence may lead to quicker fatigue [10] and increased energy expenditure [18] which is a potential barrier to motivating older adults to walk longer.

Physiotherapy and gait retraining have proven to be a positive influence on increasing stride length in older individuals [19]. However, they may not be accessible to all people as they require intensive human resources. Meanwhile, researchers have developed a partial exoskeleton that provides assistance to hip flexion. Although it was successful in increasing the step length of wearers, the device was bulky and assistance was required to put it on and off [20]. Some other devices were not portable [21] or cost-effective [20], and their applicability was not transferable to real daily activities [22]. There were devices for monitoring step length [23,24], but their focus was on gait monitoring rather than a biofeedback strategy that aims to improve gait.

While devices used in previous studies to increase stride lengths are usually bulky as they measure and control hip joint motions, another approach that has received much less attention is measuring and providing biofeedback on swing time, which is the period of time when the foot is not in contact with the floor during walking. Previous studies have reported that older adults tend to walk with shorter swing time, which may give them a sense of better walking stability [25,26,27]. However, an adequate swing phase duration is required to allow the swinging limb to move to the front with sufficient distance. A previous study demonstrated that it is the precise coordination of the lower-limb muscles to control the swing time which creates a specific distance in the step length [28]. Thin-film force resistive sensors can easily measure stance and swing time. With some light-weight biofeedback modality, users can be encouraged to increase the swing time, which may have positive effects on step lengths and the entire gait.

The use of wearable technology is an emerging field across different populations, and is a positive bridge between metrics, clinical interventions and user-accessibility in real-life settings that has been addressed in the literature [29,30]. However, among older adults, devices with practical biomechanics gait solutions that are light and portable have apparently not been developed [20,21,22]. Most wearable devices developed in the past focused on gait characterization rather than providing biofeedback in an attempt to improve gait [23,24,31].

In this study, we investigate whether a wearable device that provided haptic biofeedback to encourage lengthening of swing time could increase stride length and hip flexion, while reducing cadence. This study aims to demonstrate the potential of wearable devices in improving gait.

## 2. Materials and Methods

This was a cross-sectional study with a repeated measures design, which was used to identify whether the use of a smart insole improved gait performance in older adults. The smart insole measured the swing time and gave biofeedback through vibration to encourage users to increase the swing time by 5%. Gait analysis was performed with a focus on the four primary outcome variables (swing time, stride length, hip flexion, angle cadence). Gait mobility was assessed by the timed up-and-go (TUG) test.

### 2.1. Smart Insole System

A smart insole system was designed to track, monitor, and provide biofeedback on the gait swing time. The system is comprised of a plantar force acquisition system, as well as two feedback units.

**Figure 1:**
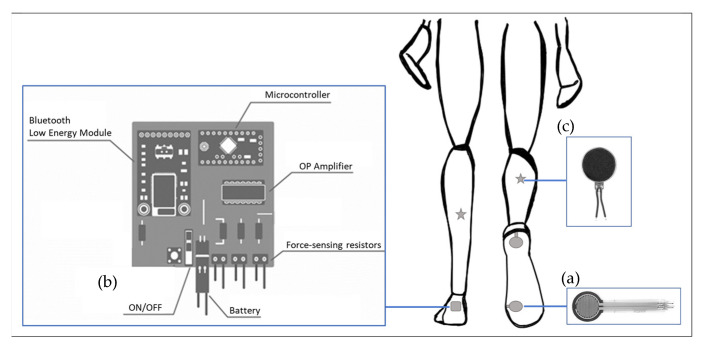
Diagram of the insole system. (**a**) Thin-film FSR placed on an insole diameter 12.7 mm, (**b**) diagram of board and electronics being host in a 3D printed box was attached at the back of each shoe, (**c**) vibrating actuator diameter 10 mm and thickness 3.4 mm place in each leg which was connected wirily to the microcontroller.

The plantar force acquisition system consisted of two thin-film polymer force sensing-resistors (18.3 mm diameter, 12.7 mm diameter sensing area, thickness 0.46 mm, and force resolution better than 0.5% full scale, FSR Interlink Electronics, Irvine, CA, USA) placed under a regular insole (Figure 1a). The force sensors were placed at the position of the calcaneus and first metatarsal head. The force sensors were used to detect the time at which the heel and forefoot touched and lifted off the ground. The sampling rate of the force sensors was 15 Hz. Bone prominence was used to identify the locations of the force sensors.

The plantar force acquisition system also included a microcontroller (ATmega328P “Arduino Pro-mini”, Atmel Co., Ltd., San Jose, CA, USA), an amplifier, a rechargeable battery (Lithium-Ion Polymer 3.7v, 350 mA), 2 wireless transmitters Bluetooth low energy modules (BT 4.1 Nordic nRF51822), and a wireless receiver (Figure 1b). A 3D printed box was designed to host the electronic elements and was attached to the back of the participant’s shoe.

The biofeedback unit consisted of two vibrating actuators (1027 Flat Coreless Vibrating Motor, Baolong Electronic Group, Yueqing, China) placed on the participant’s shank (Figure 1c). While vibrations elicit different changes in the walk depending on their location, Ivanenko et al.’s [32,33] placement method was followed, where shank muscle vibration elicited greater voluntary body displacement. Selected points were intended to avoid disruption in the walk due to hardware body position.

The vibration frequency of the actuators was 200 Hz and 1 G in strength. Each vibrator unit was activated instantly by sending a 3.5 V noise signal each time when the participant was unable to reach a customized threshold based on the participants’ gait [34]. The signal stopped once the participant was within the expected range and updated in every step.

### 2.2. Data Acquisition from the Smart Insole System

Temporal parameters were estimated based on stride segmentation and temporal intervals from the force sensing signal (Figure 1a). The swing phase is the period between the terminal contact of the foot and its successive initial contact, denominated as heel-strike (HS). For each step, the gait events identified included heel-strike (HS) and terminal contact (TC). The time between TC and HS is defined as the duration of the swing phase for each foot. Then swing phase at cycle k was computed from Equation (1), where cycle k refers to current the step, t denotes the instant when the temporal event occurred, HS^k^ considers HS at cycle k and TC^k−1^ as TC from the previous cycle k.

t^k^_swing_ = t(HS^k^) − t(TC^k−1^) (1)

The denominated swing time was collected for each leg, and a customized Labview program was used to process the signals (National Instruments Corporation, Austin, TX, USA, 2018). Each sensor was calibrated and had an individual signal threshold avoiding noise.

**Figure 2:**
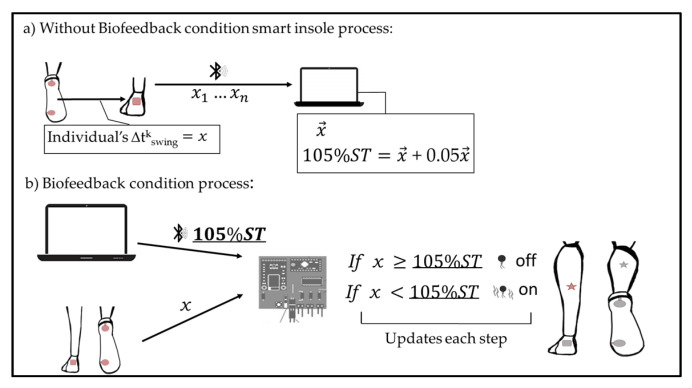
Diagram of smart insole process. Individual’s swing time (*x*) was collected from the smart insole (**a**), which would be used as a reference to increase each individual’s swing time by 5% during Biofeedback condition (**b**).

The microcontroller: (1) converted the analogue force data received from the force sensors into digital data; (2) analyzed the temporal intervals measured and then; and (3) sent a wireless control signal to the vibration unit (Figure 2).

### 2.3. Participants

The study included seven participants aged from 67 to 83 years old. There were 2 men and 5 women with body heights of 162.0 ± 10.8 cm. Five participants reported walking at least 30 min per week as a form of exercise. Participants were able to walk independently without the use of various walking devices.

Exclusion criteria were any uncontrolled cardiovascular or pulmonary diseases, cognitive impairments, history of neuromuscular pathologies, history of falling during the last year, lower limb or spine deformations, fractures or surgery in lower limbs or spine in the past two years, and severe visual dysfunction. The participants, who were recruited from the community and an independent living age care provider, performed the walking task in a flat terrain environment. Written informed consent was signed before participation. The study was conducted according to the guidelines of the Declaration of Helsinki and had Ethical Committee Human Research Ethics Committee approval, Ethic reference number 473, from the University of Wollongong.

### 2.4. Experimental Design

Each participant was tested in two conditions: With and Without Biofeedback. At both conditions, the participants were instructed to walk at their self-selected comfortable speed on a 10-meter-long flat outdoor walkway with the entire smart insole system. The vibrations were turned off in the Without Biofeedback condition (Figure 2a) and on when required during the Biofeedback condition (Figure 2b). Testing of the two conditions involved two walking sessions, which lasted altogether for about 15 min with a resting period in between. Participants walked without the biofeedback for 5 min. After resting for 5 min, they then walked with the biofeedback for 10 min. The 10 min walk allowed participants to beocme accustomed to the smart insoles with the vibration to encourage lengthening of the swing time. Gait tests were performed immediately after each walking session, in which participants walked an additional three gait trials over the 10 m walkway and, in each trial, at least three full gait cycles were recorded and analyzed. Participants were unaware of the moments when data collection was being recorded. A walking mobility test (timed up-and-go test) was conducted following the gait tests.

Average swing time was measured by the smart insole system during the Without Biofeedback condition to obtain the baseline swing time (Figure 2a). During the Biofeedback condition, the aim was to achieve an increment in the individual’s swing time. It encouraged longer swing time through vibrations depending on the swing time measured from the control condition. In the biofeedback condition, the microcontroller was set to a threshold of 105% of the average baseline swing time. Our previous test showed that participants adapted more easily to the biofeedback with a 5% increment. Participants were required to achieve an increase of a minimum of 5% in their swing time in each leg to avoid the vibrations from the smart insole system. They were unaware of the percentage by which they should increase or how they should change the entire gait to avoid the vibrations.

The Biofeedback condition was tested after the Without Biofeedback condition in each participant. Randomization of the order of the two conditions was not used, as reversing the order would increase the total test time which was not ideal for older people and might increase the risk of carry-over effects due to short wash-out time [35]. Such arrangements were also used in recent studies [36,37,38]. Each participant underwent both conditions on the same day to avoid possible day-to-day variability.

Before starting each condition, the researchers made sure that the sensors did not cause any discomfort to the participants. While placing the smart insole and vibrators, a pre-test was performed to identify whether the participant was aware of the vibrations. If a decreased sensation was present in the area, the actuator was placed above the knee on a different dermatome. Participants used their own sports shoes during the experiment.

### 2.5. Variables and Data Sources from Reference Systems

In order to investigate gait changes induced by the smart insole system, spatio-temporal variables and lower limb peak joint angles were assessed by gait analysis. Gait mobility was also evaluated by the timed up-and-go (TUG) test. The primary outcome variables were swing time (time between the last contact of the foot and the heel strike from the same foot), stride length (the distance elapsed between the first contact of two footfalls of the same foot), peak hip flexion angle (maximum hip flexion angle typically during swing phase), and cadence (number of steps per minute). The secondary outcome variables included the time measured in TUG, velocity and knee and ankle peak angles. The velocity was defined as the distance traveled by the walking time.

#### 2.5.1. Gait Data Collection

An Xsens MVN BIOMECH 3D motion capture system equipped by Xsens Studio (version 2019.0.0.0) software was used to record kinematics of the participants’ gait. The system has excellent reliability and validity assessing joint angles in sagittal plane [39]. Joint range of motion from hip, knee and ankle was collected by a 7-segment biomechanical model of lower limbs. Seven inertial sensors (3DOF wireless Motion Tracker Xsens, 47 mm × 30 mm × 13 mm, 16 g, wireless rang 50 m) were placed on the pelvis, upper leg, lower leg and foot. Joint peak angles from hip and knee flexion and extension, and foot dorsiflexion and plantarflexion were also visualised in MVN studio. Sensors were attached using customized elastic straps (Xsens Technologies B.V., Enschede, The Netherlands). Participants wore Xsens 3D motion system during the entire trial.

The GaitRite system was used to assess the spatiotemporal gait parameters (GaitRite system CIR Systems Inc., Clifton, NJ, USA). GaitRite is a standardised portable instrumented mat with an active sensing area of 61 cm wide and 488 cm long. Each sensor in GaitRite is 1.27 cm × 1.27 cm.

Both systems, Xsens and GaitRite, were synchronized, and gait data were collected with a sampling frequency of 60 Hz. The sensorized mat was placed within the 10-m-long flat outdoor walkway, ensuring that participants walked 2 m before and after the sensorized mat, avoiding deceleration and acceleration during data collection [40].

#### 2.5.2. Walking Mobility Test

The timed up-and-go test (TUG), which is a standardized clinical test frequently used in community settings [41,42], was used to inform about older adults’ mobility [43,44]. TUG provides an insight into their locomotion skills [45]. Participants stood up from a chair and walked 3 m and returned to the seated position while being timed. They walked at their self-selected comfortable speed. Lines on the floor delimited the space [46,47]. Participants had a TUG practice trial before collecting three measurements immediately after the end of each condition.

### 2.6. Data Analysis

Data were reported as the mean and standard deviation (SD) values. Data were collected from both legs and were averaged prior to statistical analyses. The normality of distribution was tested using Shapiro–Wilks tests. A Paired-sample *t*-test was used to assess whether the biofeedback device produced significant changes in the walk of older adults (without biofeedback vs. biofeedback). If the Shapiro–Wilks tests indicated that normality of data could not be assumed, the Wilcoxon test was used instead. The level of significance α was established as 0.05 [48,49]. We hypothesized that the smart insoles significantly changed the four primary outcome variables: swing time, hip flexion angle, stride length and cadence. Holm–Bonferroni method was used to reduce the family-wise error rate on these four comparisons. The probability (p) values of these four gait parameters were sorted into order from lowest to highest, and we declared significant differences only if the four *p* values (in ascending order) were below α/4 = 0.0125, α/3 = 0.0167, α/2 = 0.025, and α = 0.05, respectively.

The software GaitRite system CIR Systems Inc. computed the gait points collected before the interindividual analysis was carried out. Data points from the Xsens system were filtered in a customized MATLAB (MatLab R20 18b) code to obtain the joint peak angles of hip, knee and foot movements. Data were normalized to time by linear interpolation to 101 points. Additionally, we studied the scatter plots of the regression line of each participant in conjunction with retrievals of the modelled video provided by the software Xsens MVN BIOMECH 3D motion capture system.

## 3. Results

The seven participants included in this study had a mean age of 77.3 ± 7.3 years old. When the biofeedback was given to the participants requiring them to increase a minimum 5% of the swing time, the swing time was in fact significantly longer by 6.45%. Table 1 shows the statistically significant changes in all four primary outcome variables. With the biofeedback turned on, the cadence decreased significantly (*p* = 0.0002) by 5.51%. Meanwhile, the biofeedback significantly increased the stride length by 4.52% and the peak hip flexion angle by 14.73%.

**Figure 3:**
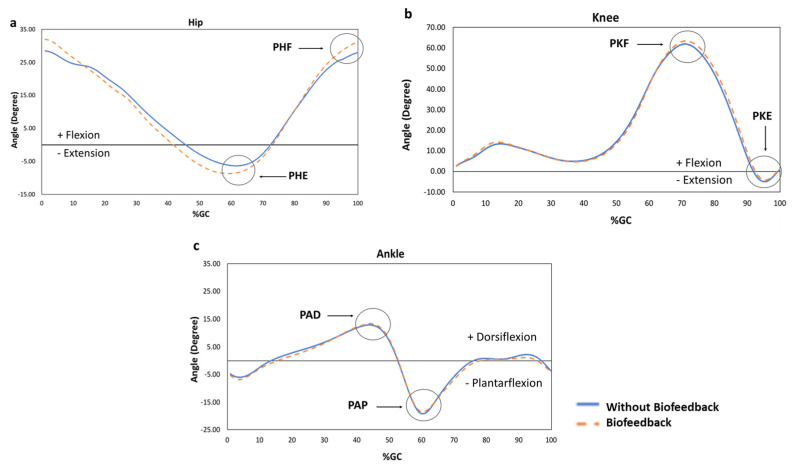
Range of motion of (**a**) hip, (**b**) knee, (**c**) ankle during the gait cycle of the dominant and non-dominant limbs, respectively, during Without Biofeedback and Biofeedback conditions. Positive values represent flexion, negative values extension. PHF, PHE, PKF, PKE = Peak hip and knee flexion and extension, respectively; PAD and PAP = Peak ankle dorsiflexion and plantarflexion. Data represents the average behavior of all participants.

In addition to the primary outcomes were the secondary outcomes variables (Table 2), which were related to the spatiotemporal and lower limb joint angles. After the biofeedback was turned on, the hip extension (Figure 3) increased by 23%, but there were no large differences in the knee ROM, ankle ROM or velocity.

The mean time completing the TUG test without the biofeedback was 9.46 ± 1.06 s. After 10 min of use of biofeedback on their swing phase, the mean TUG time reduced to 8.48 ± 1.08 s.

## 4. Discussion

The results of this study indicate that the smart insoles with haptic biofeedback that attempted to increase swing time of the gait significantly increased the swing time, and thereby significantly increased hip flexion angle and stride length while reducing the cadence significantly. These significant changes in gait parameter suggested improvements in gait.

Shorter stride length and lower speed are signs of impaired walking ability among older adults [2,50]. These changes are accompanied by a shorter swing phase [51], which creates specific adjustments in the stride duration, impacting the stride length. We found that after the biofeedback was delivered, the participants were able to swing the limbs forward over a longer period of time while increasing the stride length. This suggests that the participants were able to follow what the haptic biofeedback instructed them to do while reflecting positive changes in the gait.

While stride length is a key factor to determine walking ability [16,51], the swing phase provides momentum in the limb and facilitates the progression of the body weight [28,52]. A longer swing time comes with a greater hip range of motion, enabling the swinging limb to stride longer distances. While in this study, the instructions given to the participants allowed them to select any strategy to increase the swing time during the walk, the kinematic changes in the hip seem to be consistent with findings where older adults exhibit longer strides with greater range of motion in the hip [16,53]. These gait changes may follow Miyake et al.’s [54] notion, which suggests that during a walk, the process of acquiring new movements can be achieved through altering the range of motion of the lower limb joints. Meanwhile, the current study found that all participants increased the swing time by increasing the hip flexion during the swing, and longer stride were then recorded.

Older adults usually walk with increased cadence if they want to match the speeds of younger adults [17], which has been associated with compensation in the gait when a shorter step and lower speed is present [55]. Increased cadence could reduce the walking ability and increase the chance of early fatigue [10]. An important result of this study was a significant reduction in the cadence upon using the biofeedback. While cadence was significantly reduced by the biofeedback, the gait speed was maintained almost constant during comfortable walking and longer strides were achieved. These findings support the hypothesis that the wearable device developed in this research has promoted positive changes in the gait [10,16,56]. These results also align with the ideas of Cornwell et al. [57], who suggested that biofeedback on the temporal aspects of the gait improved spatial parameters such as step length and cadence. They also corroborate Yasuda et al.’s [58] and Choi et al.’s [59] findings, in which vibrations improved gait with longer strides.

Like many other gait analyses, we investigated many more gait parameters as well as studying mobility by using a TUG test. The participants included in this study were healthy older adults. The TUG completion time before using the biofeedback suggested that they were not at high risk of falls and their mobility was normal. However, TUG could be used as a screening tool [42]. Generally, shorter TUG time indicates higher walkability and mobility of a person [60]. In this experiment, the reduction of 10% in the completion time of the TUG immediately after using the biofeedback indicates possible improvements in the mobility of the participants. To fully assess changes in mobility and standardize the results obtained from the presented study, it is also important to include additional walking functional tests such as the 2 min walk test (2 MWT) and 10-m walk test (10 MWT) [61].

We also investigated how biofeedback changed other kinematic parameters such as knee and ankle peak joint angles. Although significant changes were only found in the peak hip flexion, a large difference was found in the peak hip extension. An increase in the hip range of motion was observed across all participants. A possible reason could have been the small sample size, as all participants increased consistently hip flexion and extension.

Biofeedback systems that provide temporal parameters of the gait in older adults are traditionally delivered in laboratories and systems that attempt to increase stride length in natural environments may encounter greater challenges. This study proved the feasibility of a wireless device that provides real-time customized haptic biofeedback to encourage users to increase the swing time during walking impacts positively on stride length and hip flexion of the gait. While most assistive devices are used indoors only, this wearable device is wireless which allows it to be used in any place.

Our healthy older participants had an improvement in stride length, hip flexion and cadence while maintaining similar self-selected speed. However, increment in swing time has a direct consequence of increasing the single support stance time of the opposite leg, which might be linked to gait stability. Future research should look into the biofeedback effects on the gait of older adults with different walking abilities with a particular focus on gait stability. Results from this research must be interpreted with caution as the participants in this study were healthy older adults. In addition, the intervention used in this study may not be suitable for older adults with balance problems as they tend to decrease the stride length and increase the cadence as functional adaptations. So far, the literature has suggested that older adults with reduced balance exhibit greater double support time [62], which may increase the proprioceptive input during the loading phase [63].

This study presents limitations to be considered in further research. Firstly, the participants in this study were healthy older adults who were able to walk independently, limiting the ability to generalize the results to other groups of older adults. Secondly, the current investigation was limited to a single session and therefore, the effects of the biofeedback among multiple training sessions are unknown. Thirdly, although this research proved potential functional changes in the ability to walk after the biofeedback was turned off, biomechanical analysis was not made after participants stopped using the device, future work should identify long-term effects. Fourthly, although vibrations have been used to increase movement performance, there is also a chance that they may cause muscle fatigue, future research should assess the presence of fatigue in a swing-time based haptic biofeedback protocol in older adults. Fifthly, the sample size was small; however, it was sufficient to detect significant differences with the level of significance set at 5% and Holm–Bonferroni method used to reduce the family-wise error rate on multiple comparisons. The small sample size was due to the health orders during the pandemic which interrupted clinical trials, particularly with older adults. Future studies should include greater sample size and a wider range of older adults.

Finally, in addition to gait analysis using XSens and Gaitrite, a clinical test of TUG was also used. Although a TUG practice trial was provided with each participant, practice effects might still affect the walking performance. We suggest that along with a greater sample size, further work should include randomized controlled trials.

## 5. Conclusions

In this study, a wireless wearable smart insole system was developed with an algorithm that based on FSR calculates the time elapsed between heel and toes and provides real-time customized haptic biofeedback. The system encourages users to increase the swing time during a walking in natural environments.

We conclude that the device significantly changed the swing time, the hip flexion angle, the stride length and the cadence of seven healthy older adults and changes in their mobility were also observed. This research inspires the use of wearable technologies to improve the walking ability of healthy older adults

## Figures and Tables

**Table 1:** Primary outcome variables in spatio-temporal parameters and peak joint angle comparing with and without the presence of biofeedback.

Parameter	Without B ± SD	95%CI	Biofeedback ± SD	95%CI	*p*-Value
Cadence	119.400 ± 7.20	112.74–126.06	112.826 ± 10.29	103.31–122.34	0.000 *
Swing Time (s)	0.38 ± 0.03	[0.37, 0.39]	0.40 ± 0.05	[0.39, 0.42]	0.000 *
Stride Length(cm)	135.75 ± 11.30	125.30–146.20	141.88 ± 12.24	130.57–153.20	0.022 *
Hip Flexion	27.1831 ± 4.08	23.40–30.96	31.189 ± 5.81	25.75–36.62	0.030 *

* with statistical significance. Values are given as the mean of the sample. Standard Deviation (SD); Without Biofeedback condition (Without B); Confidence interval (CI); Cadence (steps/min); Stride length is calculated for 2 successive footprints; T-test and Wilcoxon test with 95% adjusted. Holm–Bonferroni method was used to reduce the family-wise error rate on the four parameters. *p* values should be compared in an ascender order with α/4 = 0.0125, α/3 = 0.0167, α/2 = 0.025, and α = 0.05, respectively.

**Table 2:** Secondary outcome variables of the gait comparing with and without the presence of biofeedback.

Parameter	Without B ± SD	95%CI	Biofeedback ± SD	95%CI
Velocity (cm/s)	134.557 ± 9.44	[125.82, 143.29]	131.979 ± 12.21	[120.68, 143.28]
Norm Velocity	1.570 ± 0.184	[1.40,1.74]	1.539 ± 0.181	[1.371, 1.71]
Peak Joint angle				
Hip Extension	−11.88 ± 3.88	[−15.47, −8.30]	−14.61 ± 5.19	[−19.42, −9.81]
Knee Flexion	63.14 ± 5.17	[58.35, 67.92]	65.75 ± 8.27	[58.09, 73.40]
Knee Extension	−4.58 ± 2.52	[−6.91, −2.25]	−3.72 ± 2.52	[−7.16, −0.29]
Dorsiflexion	12.61 ± 2.82	[10.00, 15.22]	13.08 ± 2.95	[10.35, 15.80]
Plantarflexion	−20.12 ± 3.53	[−23.39, −16.85]	−20.77 ± 4.56	[−24.99, −16.55]

Values are given as the mean of the sample. Standard Deviation (SD); Without Biofeedback condition (Without B); Confidence interval (CI); Normalized velocity (Norm velocity) is the participant’s gait velocity normalized to the leg length (Leg length/s); Peak joint angles are reported in degrees.

## Data Availability

The data presented in this study are available on request from the corresponding author. The data are not publicly available due to the information contained that could compromise the privacy of research participants.
